# Long-Term Bilateral Neuromuscular Function and Knee Osteoarthritis after Anterior Cruciate Ligament Reconstruction

**DOI:** 10.3390/bioengineering10070812

**Published:** 2023-07-06

**Authors:** Payam Zandiyeh, Lauren R. Parola, Meggin Q. Costa, Madalyn J. Hague, Janine Molino, Braden C. Fleming, Jillian E. Beveridge

**Affiliations:** 1 Department of Orthopaedic Surgery, University of Texas Health Sciences Center at Houston, Houston, TX 77030, USA; payam.zandiyeh@uth.tmc.edu; 2 Department of Orthopaedics, Rhode Island Hospital/Warren Alpert Medical School of Brown University, Providence, RI 02903, USA; 3 Lifespan Biostatistics, Epidemiology, Research Design, & Informatics Core, Rhode Island Hospital, Providence, RI 02903, USA

**Keywords:** anterior cruciate ligament, reconstruction, neuromuscular function, electromyography, wavelet analysis, artificial intelligence, osteoarthritis

## Abstract

Neuromuscular function is thought to contribute to posttraumatic osteoarthritis (PTOA) risk in anterior cruciate ligament (ACL)-reconstructed (ACLR) patients, but sensitive and easy-to-use tools are needed to discern whether complex muscle activation strategies are beneficial or maladaptive. Using an electromyography (EMG) signal analysis technique coupled with a machine learning approach, we sought to: (1) identify whether ACLR muscle activity patterns differed from those of healthy controls, and (2) explore which combination of patient outcome measures (thigh muscle girth, knee laxity, hop distance, and activity level) predicted the extent of osteoarthritic changes via magnetic resonance imaging (MRI) in ACLR patients. Eleven ACLR patients 10–15 years post-surgery and 12 healthy controls performed a hop activity while lower limb muscle EMG was recorded bilaterally. Osteoarthritis was evaluated based on MRI. ACLR muscle activity patterns were bilaterally symmetrical and differed from those of healthy controls, suggesting the presence of a global adaptation strategy. Smaller ipsilateral thigh muscle girth was the strongest predictor of inferior MRI scores. The ability of our EMG analysis approach to detect meaningful neuromuscular differences that could ultimately be related to thigh muscle girth provides the foundation to further investigate a direct link between muscle activation patterns and PTOA risk.

## 1. Introduction

Anterior cruciate ligament (ACL) rupture is a prevalent knee injury that has short- and long-term consequences. Up to 250,000 ACL ruptures occur annually in the US [1,2], with those under 25 years old at greatest risk [2]. ACL reconstruction (ACLR) surgery is the current standard of care and is performed to restore functional stability while minimizing the risk of further damage to other knee structures [3]; however, most patients do not return to their pre-surgery activity levels and are at elevated risk of contralateral ACL or ipsilateral graft re-injury [4]. Patients are also likely to develop posttraumatic osteoarthritis (PTOA), despite successful surgery and post-operative rehabilitation [5,6]. The mechanisms that govern these short- and long-term risk factors remain elusive, but neuromuscular function is thought to play a role and has received a great deal of attention as it is potentially modifiable [7,8]. To this end, achieving biomechanical and neuromuscular symmetry has been the goal of many rehabilitation programs using the contralateral limb as the rehabilitation target [9,10,11,12]; however, abnormal loading, bilateral neuromuscular adaptation, and cross-over effects are present in the contralateral limb following ACL injury, which could influence contralateral limb function [13,14]. Evidence suggests that these adaptations may be attributable to changes in sensory and motor cortices following ligamentous injury [15,16,17,18]. Taken together, a localized unilateral ACL injury might have lasting central nervous system effects that manifest in bilateral changes in lower limb neuromuscular function that could theoretically contribute to the increased risk of bilateral injury [4] and long-term PTOA [19].

Surface electromyography (EMG) has been widely used to record peripheral muscle motor unit recruitment and activation characteristics during dynamic activities and is employed as a surrogate measure of underlying neuromuscular function. Differences in discrete outcome measures of EMG signal activation amplitude, onset timing, and electromechanical delay have been shown between injured and healthy patients [20], suggesting the presence of adaptive strategies. We previously employed this conventional approach to identify whether patients who had undergone ACLR at least 10 years earlier demonstrated increased quadricep/hamstring co-contraction ratios and earlier muscle activation onset compared to either their contralateral limb or uninjured controls during a one leg hop activity [21], testing the hypothesis that these measures reflect subconscious adaptive strategies to augment dynamic knee stabilization [22,23]. Contrary to our hypotheses, the discrete EMG analysis revealed that co-contraction indices were not different and that ACLR subjects demonstrated latent hamstring muscle activation onset but greater hamstring activity (as defined by area under the curve) relative to uninjured control subjects. Surprisingly, the differences occurred bilaterally, and no other significant muscle activation measures were detected despite only one-third of ACLR knees being considered clinically normal according to established International Documentation Knee Committee (IKDC) clinical exam scoring [21,24,25,26]. This discord between neuromuscular and clinical function suggested that the discrete EMG approach may be insensitive to features relevant to long-term clinical joint function. We, therefore, re-analyzed the EMG signals using wavelet analysis combined with machine learning—an approach that retains the continuous EMG signal characteristics of time, frequency, and intensity [27]. Wavelet analysis revealed that ACLR patients had, indeed, dramatically greater and prolonged normalized quadricep signal intensity and reduced hamstring intensity in their surgical leg compared to healthy, uninjured control subjects [28]. Further, the new approach identified differences between ACLR and control patients in nearly all lower limb muscles—a stark contrast to the conclusions drawn from the discrete EMG analysis approach we first employed. It remains unknown whether bilateral differences would likewise be detectible using the advanced wavelet approach, but if present, the finding would have implications for using the contralateral limb of ACLR patients as an internal control for outcome studies.

Meanwhile, the meaning of neuromuscular adaptations and their relevance to increased PTOA risk following ACL injury remain elusive. It is accepted that quadricep weakness is a risk factor for primary OA [29] and has been pursued in the setting of ACL injury where long-term functional deficits persist in hop test batteries [13]. Meanwhile, clinical, functional, and patient-reported outcomes are likewise not fully restored [24,25,26,30] and have been shown to be predictive of degenerative joint changes consistent with PTOA [24,25]. It is of note that muscle atrophy is dramatic after ACL injury and is thought to be modulated by arthrogenic muscle inhibition (AMI) [31], the origins of which are rooted in dysregulated communication between peripheral and central nervous systems [31]. If so, lasting residual muscle volume deficits resulting from prolonged muscle inhibition coupled with the presence of bilateral muscle activation changes could be an indicator of altered neuromuscular function modulated at the level of the central nervous system. We therefore speculate that prolonged muscle volume loss after ACLR could be an easily measured indicator of PTOA risk. 

Given the potential role of central nervous system involvement in long-term bilateral neuromuscular function and speculated relevance to PTOA risk in ACLR patients, we sought to: (1) identify whether ACLR wavelet muscle activity patterns were symmetrical and differed from those of healthy controls; and (2) explore whether an association exists between thigh muscle girth, worse clinical outcomes, and extent of knee PTOA in ACLR patients. We hypothesized that wavelet analysis coupled with machine learning would be sufficiently sensitive to identify bilateral differences in ACLR muscle activity patterns compared to healthy controls, and we further hypothesized that the extent of degenerative changes indicative of PTOA would be associated with reduced unilateral thigh girth and poorer clinical and functional outcomes in ACLR patients. 

## 2. Materials and Methods

### 2.1. Patient Recruitment

Twenty-three subjects were recruited from an ongoing prospective randomized controlled trial (RCT) (NCT00434837) [24,25,26]: 11 ACLR subjects (five males, six females) with a mean age of 34.7 ± 9.9 years and BMI of 27.7 ± 4.0 were seen at 11.9 ± 1.3 years post-follow-up. Twelve healthy control subjects (seven males, five females) with a mean age of 38.8 ± 6.5 years and BMI of 25 ± 3.2 were additionally recruited at the same follow-up interval. The parent RCT was designed to test whether autograft tension at the time of ACLR affected clinical, functional, patient-oriented, and OA imaging outcomes [26], which over the past decade has shown that graft tension does not have an appreciable role on these outcomes [24,25]. Patient demographics and clinical measures are shown in Table 1. For enrollment in the parent RCT, ACLR subjects had sustained an isolated unilateral ACL injury and underwent ACL reconstruction surgery using either a patellar tendon (n = 8) or semitendinosis/gracilis tendon (n = 3) autograft; subjects were excluded if they had a history of a previous knee injury, significant concomitant injury to ligaments or menisci, or demonstrated degenerative joint changes. Control subjects had no previous ligament or meniscus injuries and were matched to ACLR subject demographics at the time of original enrollment in the parent study. The index limb of the control subjects was assigned randomly to match the proportion of left versus right knee injury in the ACLR group at the time of enrollment in the parent RCT. ACLR and control subjects were invited to participate in the present sub-study if they had not sustained a second ACL or graft injury to either knee, had not sustained an intra-articular injury requiring surgical intervention, were willing to participate in all onsite examinations associated with the parent RCT protocol, the ACL graft was visible with minimal susceptibility artifact on magnetic resonance (MR) images, and females were not pregnant. A single female control subject was recruited outside of the parent study enrollment due to difficulty in recruiting from the pool of remaining female control subjects remaining in the parent RCT.

### 2.2. Study Protocol

All subjects provided written consent to participate in this Institutional Review-Board-approved study. Subjects performed a single leg hop-for-distance test during which muscle EMG signals were recorded synchronously with conventional motion capture measures (joint angles, ground contact). The hop test was selected for the dynamic activity given its application in assessing neuromuscular performance [32,33] while having excellent reliability [34,35]. Subjects strived to achieve a maximum ipsilateral hop distance without needing to take a compensatory step to regain their balance after landing. The test was repeated three times, and the average distance was calculated. The final hop distance was then reduced to 65% of the average distance to ensure subjects could land reproducibly at the center of the force plate during data collection. 

EMG activity of the following seven muscles was recorded at 3000 Hz (Desktop DTS, Noraxon, Scottsdale, AZ, USA) for three trials on each leg: Gastrocnemius Medialis (GM) and Lateralis (GL), Tibialis Anterior (TA), Vastus Medialis (VM), Rectus Femoris (RF), Biceps Femoris (BF), and Semitendinosus (ST). Electrode sites were prepared following standard EMG protocols followed by placement of bipolar Ag/AgCl EMG surface electrodes (Noraxon, Scottsdale, AZ, USA) over the muscle bellies.

The movements of the lower extremities were recorded at 125 Hz using an eight-camera optical motion capture (MoCap) system (Oqus 5+, Qualisys, Goteborg, Sweden). Ground reaction forces were collected simultaneously at 3000 Hz using a force platform (9260AA, Kistler USA, Hudson, NY, USA). MoCap and force data were low-pass filtered as detailed previously [21,28,36,37] and used only to delineate hop phases, as described in the following section.

### 2.3. EMG Post-Processing

#### 2.3.1. Signal Preparation

**Figure 1:**
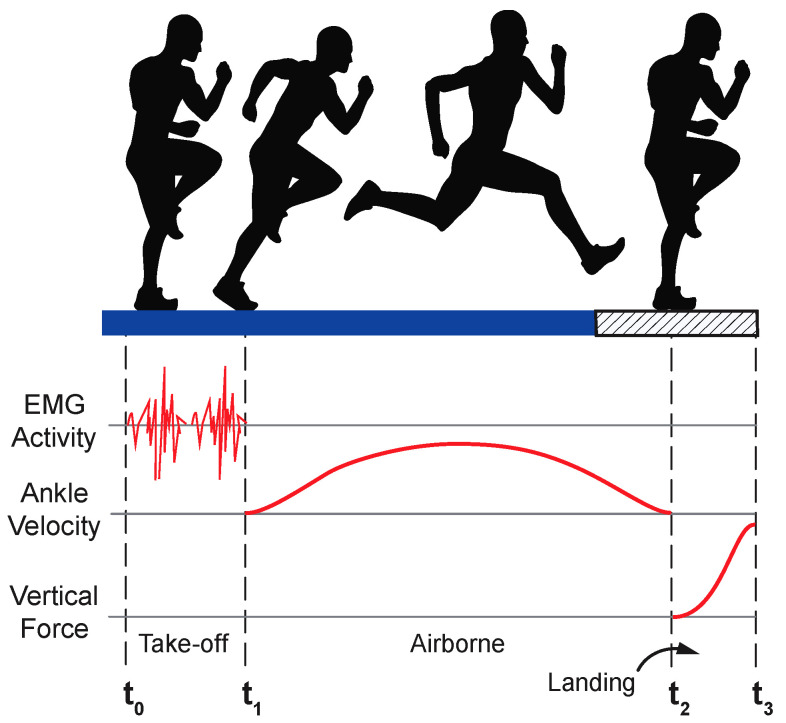
Hop phase determination. The hop activity was divided into three phases based on a combination of EMG, MoCap, and force data. t_0_ = start of trial based on increased EMG signal amplitude; t_1_ = take-off based on vertical ankle velocity; t_2_ = contact based on ground rection force; and t_3_ = peak vertical ground reaction force. Figure adapted from [28].

Based on visual inspection, EMG signals were free of motion artifacts or other spurious occurrences. Hop test data were subdivided into three distinct phases based on a combination of EMG, MoCap, and force data (Figure 1): *1. Take-off:* time zero (t_0_) was considered to have occurred when EMG activity and knee flexion increased from near-zero baseline EMG signals and static MoCap data values. The end of the take-off phase (t_1_) was defined as the minimum ankle velocity in the vertical direction before force plate contact; *2. Airborne:* spanned from lift-off (t_1_) to contact (t_2_), where t_2_ was the instant that the vertical ground reaction force exceeded 50 N; *3. Landing:* spanned from initial contact (t_2_) to peak ground reaction force (t_3_). 

#### 2.3.2. Signal Conditioning and Generation of Wavelets

First, the AC power line frequency (60 Hz) and its harmonics were removed from EMG signals using a notch filter. Next, signals were band-pass filtered within a 7–700 Hz window [38]. Finally, a filter bank of 14 non-linearly scaled Gaussian wavelets was then applied to the EMG signals to calculate the muscle activity patterns. The 14 central frequencies of the filter bank were selected because they encompassed the full range of muscle activity frequencies observed in this study [27,28].

#### 2.3.3. Wavelet Normalization and Visualization

Following wavelet generation, data were subdivided into the three hop phases and normalized to 100 timeframes for each phase. To remove baseline differences in EMG intensity between subjects, the mean signal intensity was subtracted from the original signal and then divided by its standard deviation. This normalization was performed for each of the wavelet frequency bands at each time frame of data—i.e., 100 timeframes for each hop phase. Following normalization, EMG intensities (in mV/mV) from the three trials and across subjects could be averaged.

**Figure 2:**
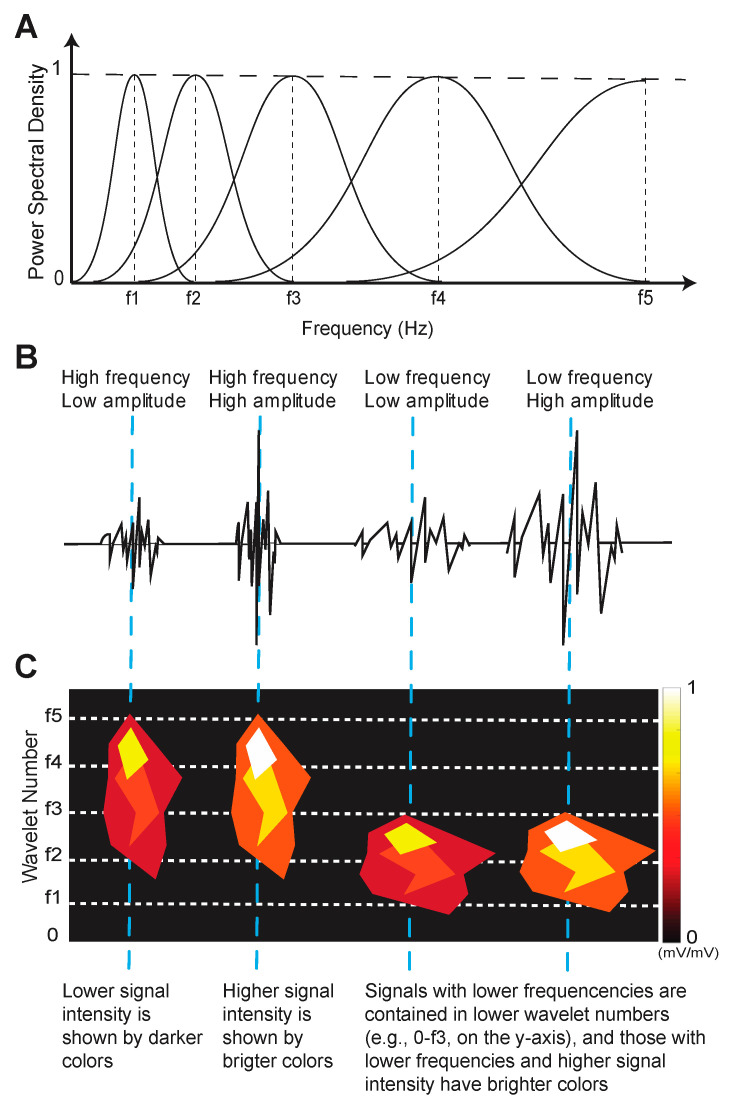
Example of EMG wavelet visualization with 5 frequency bands f1–5. (**A**). EMG signals are band-pass filtered according to their central frequency (vertical dashed lines). (**B**). Signal frequencies and amplitudes determine the signal intensity. (**C**). Examples of wavelet objects that represent frequency content (e.g., height of shape according to y-axis), intensity (e.g., color), and time (e.g., shape width according to x-axis). The dashed blue lines that span (**A**–**C**) illustrate how the combination of wavelet components is represented in a wavelet object. Figure adapted from [28].

Figure 2 describes how to interpret the visual representation of the EMG signal frequency wavelets. The height and vertical shape of the object represents the frequency content of the wavelet pattern and corresponds to the y-axis frequency range; normalized signal intensity relates to EMG signal amplitude and is color-coded according to the color bar; the width and placement of the EMG signal wavelet object and its corresponding frequency bands along the x-axis correspond to its occurrence in time. 

#### 2.3.4. Machine Learning Classification

The k-Nearest neighbors (k-NN) machine learning algorithm (k = 1) was employed to classify muscle activity patterns as belonging to ACLR or Control based on the EMG signal time, frequency, and intensity content, as described previously [28]. Vectorized muscle patterns were inputted into the k-NN algorithm four times: once for an analysis that included all hop phases and a separate analysis corresponding to each of the three hop phases. A leave-one-out cross-validation method was applied to test the classification accuracy and was chosen to accommodate the small dataset [39]. We have previously shown that ACLR surgical limb wavelet muscle activity patterns are different from control index limb patterns [28]. We therefore performed three pair-wise comparisons to enable hypothesis testing for the current study: (1) Control subject index vs. contralateral limb muscle activation patterns to establish bilateral symmetry in healthy controls; (2) ACLR surgical vs. contralateral patterns to determine the extent of bilateral symmetry present in ACLR subjects; and (3) ACLR contralateral vs. Control index patterns to determine the extent that ACLR contralateral limb muscle activation patterns are different from those of controls’. The nomenclature for these comparisons and their abbreviations are described in Table 2.

#### 2.3.5. EMG Statistical Approach

A binomial distribution test was used to test whether the classification of muscle activity patterns was significantly different for pair-wise comparisons. Given a sample size of n = 23, *p* < 0.05, and 80% power, the critical classification rate was 65.2%, which was then adjusted to 73.9% following a Bonferroni correction for multiple comparisons (p_adjusted_ = 0.008). Therefore, if correct k-NN classification occurred at a rate greater than 73.9% of the time, differences in muscle activation patterns were considered statistically significant. Our statistical power for the correct classification rate using the machine learning approach was directly related to sample size (65.2%, n = 23) and the number of comparisons being made (65.2% adjusted to 73.9% with three comparisons), as described above.

### 2.4. Muscle Girth, Clinical, Functional, and Patient-Reported Outcomes

Side-to-side differences in thigh muscle girth were determined by measuring thigh circumference 6 cm above the knee joint line bilaterally and expressing the final measurement as the difference from contralateral to the nearest +/−0.5 cm. Clinical, functional, and patient-reported outcome measures included: (1) knee laxity; (2) hop distance; and (3) Tegner activity scores, respectively. These outcomes were selected based on their sensitivity to demonstrate persistent deficits in ACLR patients compared to matched healthy controls [24,25,26]. *(1) Laxity:* anterior–posterior laxity (in mm) was measured using an arthrometer (KT-1000, Medmetric, San Diego, CA, USA); (*2) Hop Distance:* hop distances of the surgical/index and contralateral limbs were expressed as a percentage: (ACLR÷Contralateral) × 100; (*3) Tegner:* Tegner activity scores were determined by grading work and sports activity levels.

### 2.5. Knee PTOA Score

The extent of degenerative changes consistent with PTOA was assessed in both ACLR and control subjects through MR imaging using the Whole Organ Magnetic Resonance Imaging (WORM) score [40] according to the protocols of the parent RCT [24,26]. The images were scored by a musculoskeletal radiologist blinded to the limb side and study cohorts. MR scans were acquired bilaterally with a 3.0 Tesla PRISMA whole-body scanner and a 16-channel circumferential coil (Siemens Inc., Munich, Germany). The details of the MR imaging sequences are provided in Table A1 of Appendix A. WORM scores were based on 14 independent features related to PTOA, cartilage signal and morphology, sub-articular bone marrow abnormality, sub-articular cysts, sub-articular bone attrition, and marginal osteophytes evaluated across 15 regions. The condition of menisci, cruciate and collateral ligaments, synovitis, loose bodies, and periarticular cysts was also included. All WORM sub-scores were summed to create a single score representative of PTOA status for each knee. 

### 2.6. Correlation and Regression Analyses

The presence of significant correlations between knee PTOA features and clinical and functional outcomes (thigh girth, laxity, hop distance, and Tegner activity scores) was tested using two-tailed Pearson tests. A stepwise linear regression analysis was then performed to identify which clinical/functional outcomes (independent variables) significantly predicted the ACLR surgical PTOA score (dependent variable). All statistical comparisons were performed using IBM SPSS Statistics (Version 26, IBM Corp., Armonk, NY, USA).

## 3. Results

### 3.1. Muscle Activity Pattern Classification

**Figure 3:**
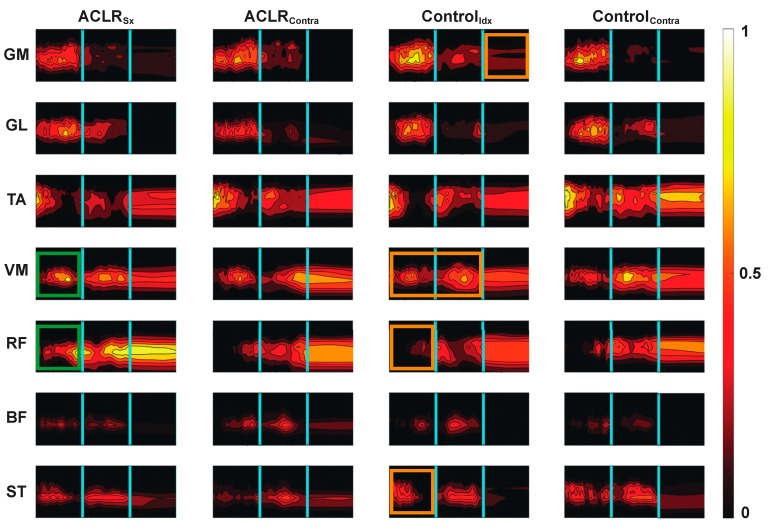
Average wavelet muscle activity patterns. The horizontal axis represents each activity phase’s normalized time. The y axis in each figure represents the activation frequency (0 to 1000 Hz), and the contour intensity is the normalized activity intensity scaled from 0 to 1 mV/mV with the brighter colors representing a higher relative activation intensity. The cyan vertical lines delineate take-off, airborne, and landing hop phases. Significantly classified patterns between the ACLR_Sx_ and Control_Idx_ limbs [28] and ACLR_Sx_ and ACLR_Contra_ (e.g., Table 4b)) are highlighted by orange and green boxes, respectively. GM: Gastrocnemius medialis, GL: gastrocnemius lateralis, TA: tibialis anterior, VM: vastus medialis, RF: rectus femoris, BF: biceps femoris, and ST: semitendinosus.

Average wavelet muscle activity patterns are shown in Figure 3. When the one-leg hop activity was considered as a whole, healthy control subjects demonstrated symmetrical muscle activity patterns as demonstrated by correct classification rates that ranged from ~35 to 56% (Table 3, comparison 1). Apart from the tibialis anterior, ACLR muscle activity patterns were also bilaterally symmetrical (Table 3, comparison 2). Conversely, contralateral limb ACLR muscle activity patterns were statistically different from control patterns with correct classification rates ranging from ~78 to 94% (Table 3, comparison 3).

The ACLR classification results for each separate hop phase are shown in Table 4. Quadricep muscle activity patterns of the ACLR surgical limb during take-off were different from contralateral limb patterns (Table 4a)), which appear to be due to increased normalized intensity (Figure 3, green boxes). ACLR hamstring activity was significantly different from controls’ during the airborne phase (Table 4b)), which appears to be driven by shorter but more intense activity based on the visualization of Figure 3. With the exception of the tibialis anterior, landing classification rates showed the lowest correct rates, ranging from ~46 to 70%.

### 3.2. Muscle Girth, Clinical, Functional, and Structural Correlations

WORM scores of the index limb were more variable in ACLR subjects (SD = 24.5) compared to Controls (SD = 4.9). Inferior WORM scores correlated with smaller muscle girth (R^2^ = −0.74, *p* = 0.009) and greater hop distance asymmetry (R^2^ = 0.65, *p* = 0.032) in ACLR subjects. Also, a trend was observed between WORM scores and hop distance asymmetry (R^2^ = −0.56, *p* = 0.073). The stepwise linear logistic regression model revealed that smaller muscle girth was the only outcome significantly associated with inferior WORM scores (B_0_ = −8.3, ß= −20.8, *p* = 0.009).

## 4. Discussion

Our results demonstrate that ACLR patients had bilaterally symmetrical muscle activity patterns. When interpreted alongside our first wavelet EMG investigation that examined only ACLR surgical limb vs. healthy control index limb muscle activation patterns [28], we can conclude that the muscle activation patterns of *both* ACLR limbs are different from those of healthy controls more than a decade post-ACL reconstruction surgery. The second major conclusion of this study is that smaller ipsilateral thigh muscle girth was associated with the presence of greater knee degeneration consistent with PTOA onset.

When we employed conventional discrete EMG measures, we found only subtle hamstring functional differences between ACLR limbs and controls [21]. Contrary to this finding, the combination of wavelet analysis and a machine learning method revealed dramatic differences in lower limb muscle activation patterns across several muscle groups in addition to hamstrings, with greater normalized quadricep activation being particularly notable [28]. The present study adds new knowledge to this earlier finding in showing that contralateral limb muscle activation patterns were more ACLR-like than those of healthy controls, and that they could be considered permanent as they were present over 10 years after initial injury and surgery. While the convergence towards kinematic and kinetic symmetry by 2 years has been described [41], to our knowledge, the presence of bilateral EMG symmetry has not been described in this clinical population at long-term follow-up, nor investigated using such a sensitive tool that can be applied during a dynamic task like the one-leg hop test. The presence of bilateral muscle activation symmetry may have implications for identifying how the nervous system adapts to ACL injury and surgery and its plasticity over time if contralateral limb muscle activation patterns become more ACLR-like in the absence of structural injury, which could only occur if changes are occurring at the central nervous system level [18]. 

When we investigated the hop activity as a whole (e.g., Table 3), our observation of bilateral symmetry in ACLR dovetails the body of work that has described bilateral decreases in voluntary quadricep activation [42,43], altered muscle pattern recruitment [44], and inferior central activation ratios [45] at earlier (<2 yrs) post-operative time points that do not fully recover [46]. Further, there is evidence that these symmetrical measures may even progress in abnormality with time, as compared to healthy control measures [47]. Interpreted alongside the pre-existing literature, our results bring into question the validity of using the contralateral limb as an internal control in the ACL-injured population. At the same time, asymmetries in quadricep motor activation [45] and hip muscle (gluteus maximus and medial hamstring) EMG amplitudes [44,48] have been noted in some ACLR patients to suggest that the involved limb remains more abnormal than the contralateral, even if neither limb can be considered neuromuscularly “normal”. Although our results of the paired comparison between ACLR limbs did not reach significance, except for TA (Table 3, comparison 2), the correct classification rates that ranged from 66 to 71% for the contralateral limb ACLR muscle activation patterns neared the critical classification rate of 73% and would have been considered significant prior to Bonferroni adjustments. This observation suggests that the underlying neuromuscular function of the contralateral limb may be somewhere between the involved limb and healthy controls, which would align with the observations in voluntary quadricep activation made by Urbach and colleagues [46]. A larger sample size would be necessary to confirm this speculation empirically. Meanwhile, caution is advised in the use of the contralateral limb as an internal control when assessing neuromuscular function.

When the hop activity was sub-divided and evaluated by hop phase (Table 4), much of the sensitivity to detect changes was lost. The higher, although not statistically significant, correct classification rates during the planning and airborne phases suggest that these two phases contributed most to the machine learning algorithm sensitivity when the whole hop activity was evaluated. Even when evaluated by hop phase, the ACLR contralateral hamstring activity during the airborne phase was significantly different from controls (Table 4b) and was characterized by latent activation but more prolonged activity spanning all three hop phases, particularly for the semi-tendinosis, as seen in Figure 3. These differences align with the greater hamstring EMG area under the curve we first reported in Behnke et al. [21] and could reflect a pre-programmed strategy to augment dynamic joint stability prior to landing [18,49] as the hamstrings function as ACL antagonists [50]. 

In an effort to discern the relevance of these muscle activation differences in the context of long-term PTOA risk, we sought to explore which clinical, functional, and patient-reported outcome measures associated more strongly with knee PTOA scores, including elements associated with neuromuscular function—hop distance and muscle girth. The stepwise linear regression revealed that only muscle girth was a significant factor associated with degenerative changes consistent with PTOA onset. Although we cannot say whether reduced muscle girth was a direct result of the different muscle activation patterns we observed in ACLR patients, the literature describing reduced motor cortex excitability [28] and compromised response to external perturbations [51,52] following ACLR suggests that prolonged mal-adaptations may be sufficient to induce widespread muscle activation disturbances that could explain muscle weakness and atrophy, which are known risk factors for symptomatic primary knee OA [53]. Detecting the association between muscle girth and elevated PTOA scores in the current work is significant as it contributes to our understanding of the natural history of PTOA with reduced muscle volume playing a role and being potentially modifiable [14]. 

While the ACLR patients demonstrated statistically symmetrical classification rates in their muscle activation patterns, the regression models focused on the surgical limb which tended to have higher PTOA scores than the contralateral joint scores. These results from the current subset of patients align with our most recent analyses of the larger parent study dataset (n = 47 ACLR; n = 26 Control) which has demonstrated that ACLR patients have significantly more degenerative changes consistent with PTOA compared to matched controls at 10–12-year follow-up [25]. While we and others [54] speculate that muscle activation patterns and neuromuscular function contribute to PTOA in some way, it is highly likely that pro-inflammatory cascades at the time of injury and surgery also contribute to PTOA pathogenesis [55] and could explain the unilateral differences in PTOA scores. “Primed” by the exposure to pro-inflammatory cytokines, a small change in neuromuscular function and its downstream effects on dynamic joint contact mechanics [56] could be sufficient to promote articular cartilage degradation. Conversely in the contralateral limb, tissues may be able to withstand and adapt to the small and more gradual shift in dynamic function. Nonetheless, the role of contralateral limb neuromuscular plasticity with respect to increased injury risk [18] and long-term joint health is a topic of ongoing investigation.

One limitation of the current study is its sample size: with only 23 subjects (11 ACLR patients), the scope of inference is limited and is commensurate with a pilot study. Nevertheless, significant differences between patient groups were detected using a conservative approach, suggesting that a Type II error did not occur and supports the sensitivity of the methods used in this work. Secondly, additional potential sources of variability in our findings could be the graft type and/or graft tension applied at the time of implantation; however, data from the parent study [24] and others [30] suggest that any differences due to graft type are inconsequential 10–15 years post-ACLR. The exception to this is that male subjects who received a “low-tension” autograft demonstrated worse radiographic scores at their 10–12-year follow-up [25]. Because the current ancillary study involved only a subset of the parent RCT subjects, we do not have adequate statistical power to explore the main and/or interaction effects of graft tension and sex on neuromuscular function and PTOA risk at this time. Thirdly, while a repeatability analysis was not performed, the EMG measurements are reasonably repeatable since: (a) standardized methods of EMG placement and recording were practiced, and (b) EMG recordings during similar movements (e.g., hurdling, jumping, cutting) have been shown to be reliable and reproducible [57]. Lastly, we do not know the timing of contralateral limb muscle activation changes and their potential response to targeted intervention, which could be the topic of future studies.

## 5. Conclusions

Our results provide insights into the bilateral nature of neuromuscular abnormalities at long-term follow-up after ACL reconstruction and the potential role of neuromuscular abnormality in modulating PTOA risk. We provided additional evidence that our EMG signal wavelet analysis approach is sufficiently sensitive to muscle activation pattern abnormalities that may be beneficial from a joint stability standpoint, but maladaptive in terms of long-term joint health if ultimately related to muscle mass. Lastly, thigh muscle girth—more so than clinical, functional, and patient-reported outcomes—was strongly associated with MRI-based PTOA scores, providing additional evidence that neuromuscular function may contribute to long-term PTOA risk. 

## Figures and Tables

**Table 1:** Subject demographics and clinical outcome measures at follow-up.

Sex	Subject Group	Age	Index Limb	BMI	Follow-Up Year	Tegner Score	KT-1000 ^a^	Hop Distance ^b^
Male	ACLR	27	L	25	12	6	−1	94
Male	ACLR	29	L	33	12	3	3	85
Male	ACLR	30	L	29	12	9	−2	93
Male	ACLR	30	R	26	12	6	0	84
Male	ACLR	27	R	27	10	5	1	113
Female	ACLR	31	L	22	15	6	1	100
Female	ACLR	39	R	29	12	6	0	97
Female	ACLR	29	R	32	12	5	−2	85
Female	ACLR	36	R	20	12	7	0	104
Female	ACLR	60	L	26	12	4	−14	108
Female	ACLR	44	L	28	10	6	3	95
Male	Control	33	R	27	12	5	0	108
Male	Control	34	L	27	12	4	0	95
Male	Control	41	L	26	12	5	1	100
Male	Control	35	L	26	12	7	−1	95
Male	Control	31	R	20	10	6	−1	101
Male	Control	47	L	24	12	7	0	94
Male	Control	31	L	31	12	3	−1	111
Female	Control	43	L	21	12	6	1	96
Female	Control	38	R	26	10	6	0	106
Female	Control	49	R	23	15	6	0	91
Female	Control	45	R	21	12	6	−1	100
Female	Control	26	L	22	N/A	4	0	91

^a^ Difference from contralateral limb in mm; positive values indicate increased laxity. ^b^ Difference from contralateral limb in %; >100 indicates greater than contralateral.

**Table 2:** Numbered pair-wise comparisons performed and nomenclature of comparison.

Comparison	Variable Names
Between index (Idx) and contralateral limbs in Control subjects	Control_Idx_ vs. Control_Contra_
2. Between surgical (Sx) and contralateral (Contra) limbs in ACLR subjects	ACLR_Sx_ vs. ACLR_Contra_
3. Between ACLR contralateral and Control index limbs	ACLR_Contra_ vs. Control_Idx_

**Table 3:** k-NN classification rates for entire hop activity. Bolded results indicate significance based on a critical correct classification rate of 73.9%. Nomenclature for the comparisons is described in Table 2. GM: Gastrocnemius medialis, GL: gastrocnemius lateralis, TA: tibialis anterior, VM: vastus medialis, RF: rectus femoris, BF: biceps femoris, and ST: semitendinosus.

		(1) Control_Idx_ vs. Control_Contra_	(2) ACLR_Sx_ vs. ACLR_Contra_	(3) ACLR_Contra_ vs. Control_Idx_
		Classification (%)	Classification (%)	Classification (%)
Muscle	GM	34.8%	69.4%	**77.8%**
	GL	55.1%	69.4%	**82.5%**
	TA	44.9%	**75.8%**	**90.5%**
	VM	49.3%	66.1%	**85.7%**
	RF	56.5%	69.4%	**77.8%**
	ST	40.6%	69.4%	**88.9%**
	BF	47.8%	71.0%	**93.7%**

**Table 4:** k-NN Classification results by hop phase for ACLR comparisons: (**a**) ACL_Sx_ vs. ACLR_Contra_; (**b**) ACLR_Contra_ vs. Control_Idx_. Bolded results indicate significance based on a critical correct classification rate of 73.9%. The nomenclature for the comparisons is described in Table 2. GM: Gastrocnemius medialis, GL: gastrocnemius lateralis, TA: tibialis anterior, VM: vastus medialis, RF: rectus femoris, BF: biceps femoris, and ST: semitendinosus.

(a)	ACLR_Sx_ vs. ACLR_Contra_		
	Take-off	Airborne	Landing
GM	64.5%	58.1%	46.8%
GL	67.7%	64.5%	56.5%
TA	72.6%	72.6%	58.1%
VM	**75.8%**	62.9%	51.6%
RF	**77.4%**	62.9%	46.8%
BF	67.7%	62.9%	62.9%
ST	61.3%	67.7%	58.1%
(b)	ACLR_Contra_ vs. Control_Idx_		
	Take-off	Airborne	Landing
GM	63.5%	63.5%	58.7%
GL	**82.5%**	66.7%	55.6%
TA	69.8%	66.7%	**81.0%**
VM	68.3%	73.0%	50.8%
RF	63.5%	63.5%	46.0%
BF	58.7%	**82.5%**	68.3%
ST	71.4%	**77.8%**	69.8%

## Data Availability

The data are not publicly available but may be made available upon reasonable request to the corresponding author.

## Appendix A

**Table A1:** MR imaging scan sequences used for knee PTOA scoring.

Sequence
Sagittal T1-weighted water-excitation three-dimensional (3D) fast low-angle shot (3D FLASH): 20/7.6 [TR msec/ TE msec]; 12° [flip angle]; 160 mm [field of view, FOV]; 1.5 mm/0 [slice thickness/interslice gap]; 80 slices per slab; 130 hz/pixel [bandwidth, BW]; 512 × 512 [matrix]; right/left [phase encoding axis]; one average of two excitations.
Coronal Intermediate-weighted turbo-spin echo (TSE): 3850/29; 7 [echo train length, ETL]; 140 mm; 3 mm/0 mm; 41 slices; 352 hz/pixel; 307 × 384; right/left; one average.
Sagittal ^†^ T2*-weighted WE-3D double echo steady state (WE-3D DESS): 16.3/4.7; 25°; 140 mm; 0.7 mm/0 mm; 185 hz/pixel; 307 × 384; anterior/posterior; one average.
Sagittal Intermediate-weighted TSE with fat-saturation: 3460/36; 5 ETL; 160 mm; 3 mm/0 mm; 248 hz/pixel; 314 × 448; superior/inferior; one average.

^†^ Additional MR images were reconstructed in the axial and coronal planes.
